# Nurses’ Views on Caring for Lonely Patients During the Pandemic: A Qualitative Study

**DOI:** 10.7759/cureus.57639

**Published:** 2024-04-05

**Authors:** Areti Stavropoulou, Margarita Daniil, Georgia Gerogianni, Georgios Vasilopoulos, Georgia Fasoi, Dimitrios Papageorgiou, Chrysoula Dafogianni, Martha Kelesi

**Affiliations:** 1 Department of Nursing, Univeristy of West Attica, Athens, GRC; 2 Department of Nursing, University of West Attica, Athens, GRC

**Keywords:** qualitative study, covid-19, visitation restrictions, family, lonely patient, nursing care

## Abstract

Background: The implementation of safety measures in hospitals due to the COVID-19 pandemic, including family visitation restrictions, forced the family to be absent during the patient’s hospitalization. Nurses were confronted with new roles and responsibilities, as caring for lonely patients was a new phenomenon that emerged during the pandemic.

Purpose: The purpose of this study was to explore the nurses’ views on caring for lonely patients during the COVID-19 pandemic.

Methods: A qualitative research method based on an inductive content analysis approach was used. Data collection was carried out using semi-structured interviews. The sample consisted of 11 nurses who worked in COVID-19 wards and units. Content analysis was used to analyze the interview data.

Results: The analysis of the data revealed three main themes: 1) caring for the patient and the family; 2) extending conventional care; and 3) developing supportive mechanisms for better care. Caring for lonely patients brought about changes in patients’s management and care and significantly affected communication patterns as well as nurses’ roles and responsibilities. Providing care beyond limits, supporting care through the utilization of new technologies, and transforming organization and care were mentioned as areas of challenge for nursing practice.

Conclusions: Maintaining communication and supporting the family’s involvement in patient care are considered to be equal to healing for the lonely patient. Reshaping working conditions and caring practices to meet the needs of the nurses, the patients, and their families during health crises may contribute positively to the provision of holistic care for patients and their families.

## Introduction

In December 2019, the COVID-19 virus was identified as a rapidly transmitting respiratory disease in the city of Wuhan, China, with more than 81,000 confirmed cases across the country. In March 2020, the World Health Organization (WHO) declared the disease a pandemic as its transmission had spread throughout Europe and the United States [1]. The COVID-19 outbreak affected all countries economically, politically, and socially, causing harmful effects on the quality of life of people around the world [2].

Several changes occurred in healthcare systems internationally, due to the increased rate of hospital admissions and the unpreparedness of the health services to cope with an unknown and life-threatening virus. Healthcare professionals faced a rapidly changing environment and the adaptation of novel protocols that greatly influenced the caring and clinical practice processes [1]. One of the many changes implemented in hospitals due to the pandemic was the restriction of family visits for COVID-19 patients. The limited involvement of the family in the patient's care and the visiting restrictions caused additional pressure on the already overburdened healthcare professionals, who have reported negative emotions such as fear, denial, social exclusion, and depression [3,4]. On the other hand, COVID-19 patients conveyed feelings of loneliness, isolation, and abandonment, which were caused by separation anxiety from their loved ones [5]. Healthcare professionals, especially nurses, committed themselves to additional roles and responsibilities due to the absence of the family, such as facilitating patient-family communication via information technology (IT) media or acting as the mediator between the “absent” family and the dying patient [6-8].

Relevant literature reports that critical issues caused by the restriction visitation policies, such as the patients who were dying alone without the presence of their families, deeply affected healthcare professionals, who described these cases as the darkest experiences of their professional careers [9]. Nurses who provided end-of-life care during the pandemic experienced feelings of empathy, compassion, mourning, and psychological exhaustion and characterized the absence of the family as one of the most difficult parts of their work during the pandemic [10]. However, in cases where face-to-face contact between family and patient was exceptionally scheduled, nurses felt relieved and emotionally discharged [11]. During the pandemic, nurses were identified as the professionals with the greatest physical and emotional burden and yet as those who remained committed to providing high-quality care to their patients [2, 12, 13].

Although several studies in Greece explored issues related to nurses’ psychological burden, stress, burnout, satisfaction with providing information, and experience of caring for COVID-19 patients [13-15], research evidence on the phenomenon of caring for lonely patients due to family visitation restrictions is rather limited. This study aimed to explore the nurses’ views and perceptions of caring for lonely patients during the COVID-19 pandemic. The evidence derived from this study is expected to contribute to the development of knowledge in the field under investigation and further enrich the relevant scientific evidence.

## Materials and methods

In this study, a qualitative research approach based on the principles of inductive content analysis was used [16]. Qualitative research approaches focus on the description and understanding of phenomena and human experiences within the social context in which they occur [17]. These methods have a leading role in the health sciences, as they focus on an in-depth understanding of human views, perceptions, and experiences and attempt to explore issues that have not been sufficiently studied [18]. As the aim of our study was to explore nurses’ views on caring for lonely patients during the COVID-19 pandemic, the qualitative research approach was considered the most appropriate one.

Sample and sampling strategy

Nurses who worked in a COVID-19 referral hospital and were exclusively involved in the care of hospitalized COVID-19 patients constituted the study population. Additional inclusion criteria involved: a) registered nurses holding a university degree; and b) working in a COVID-19 ward or unit for a period of at least six months. This specific period of work experience with COVID-19 patients was considered essential for the participants to be able to offer a wealth of information on the topic under investigation. Nursing assistants were not included in the study.

A purposeful sampling strategy was used. Recruitment of the participants was carried out by one member of the research team, who advertised the study through informal meetings with the nurse managers and the nursing staff of the hospital. Potential participants were informed about the nature and purpose of the study as well as the inclusion criteria for their participation in it. The researcher provided full access to relevant contact information so that nurses who wished to participate in the study could be more precisely informed about participation details. Eleven nurses who fulfilled the inclusion criteria joined the study.

Data collection

Semi-structured, face-to-face interviews were used for data collection purposes. In qualitative studies, interviews are recommended as the most common way of collecting data, while semi-structured interviews are the tool of choice for many qualitative researchers [19]. 

The interview plan for the present study included four open-ended questions regarding the views and perceptions of nurses about a) caring for COVID-19 patients amid the family visitation restriction; b) communication and family involvement in patient care; c) nurses’ role in patient and family support; and d) supporting mechanisms for family and COVID-19 patients due to visitation restriction policies.

The interviews were conducted by a female nurse, an experienced member of the authoring team who had received appropriate training on how to perform and complete the interviewing process. The participants were approached by the researcher before the commencement of the interviews to be informed about the research process. There was no professional interaction between the researcher and the participants that might influence the data collection and interpretation. The interviews were conducted at a location and time chosen by each participant from April to June 2022. Attention was given to the selected location to ensure confidentiality and avoid any form of interruption during the interview process. A tape recorder was used following each participant’s consent. The interviews lasted from 15 to 35 minutes. A total of 11 interviews were conducted. Data saturation was achieved as, after the ninth interview, no new data emerged, which might lead to the generation of new codes and themes.

Data analysis

Interview data were analyzed following the basic principles of inductive content analysis, which include the phases of data reduction, data grouping, and the generation of concepts [16]. During the data analysis process, the researcher applied specific actions, e.g., repeated reading of the interview transcripts, coding, and categorization of the data, and comparing similarities and differences between coded data for organizing and further developing concepts, categories, and themes. [16,19,20]. The review and refinement of the initially formulated themes led to the development of the final themes and sub-themes, which illustrated the essence of the collected data and provided answers to our study topic.

Ethics

Study participants were fully informed about the nature and aim of the research. Protection of personal data, confidentiality of data, and anonymity were strictly preserved. Participants were informed about their right to withdraw from the study at any stage of the research process without any consequence. They were also assured that the interview data would be used exclusively for scientific purposes, the presentation of the data would not include any identification information, and full access to the study findings may be granted to them upon request. Before the commencement of the study, an informed consent form was signed by each participant. Research approval was granted by the Scientific Committee of General Hospital of Athens "Sotiria", Athens, Greece (ref. no. 7144/18-3-2022).

Trustworthiness of the study

Reflexivity and triangulation were applied to ensure the trustworthiness of the present study [21]. In particular, reflexivity involves the examination of the researcher’s personal values, perceptions, and biases through the use of a study diary. This strategy enabled the researcher to control particular preconceptions or assumptions and identify how these may affect decision-making at all stages of the investigation. Triangulation involved a second analyst who examined the coding, the analysis, and the interpretation of the data to ensure the reliability of the findings. The consolidated criteria for reporting qualitative research (COREQ) were applied [22].

## Results

The sample of the present study consisted of 11 registered nurses, and university graduates who worked in COVID-19 wards and units with a minimum work experience of seven months. The demographic data of the study participants are presented in Table 1.

**Table 1:** Demographic data of the study participants P: participant

Participant code	Gender	Age	Marital status	Working experience with COVID-19 patients	Working experience (total)
P1	F	30	Single	2 years: COVID unit	5 years
P2	F	47	Married	2 years: COVID ward	23 years
P3	M	40	Single	2 years: COVID ward	15 years
P4	F	40	Married	15 months: COVID ward	12 years
P5	F	42	Married	2 years: COVID ward	18 years
P6	F	38	Married	2 years: COVID ward	6 years
P7	F	40	Single	2 years: COVID ward	12 years
P8	F	42	Married	2 years: COVID ward	19 years
P9	F	30	Single	2 years: COVID unit	20 months
P10	M	38	Married	2 years: COVID unit	7 years
P11	M	36	Married	7 months: COVID unit	7 months

The analysis of the data revealed three main themes and seven sub-themes, as illustrated in Table 2.

**Table 2:** Main themes and sub-themes of the study

Main themes	A. Caring for the patient and the family	B. Extending conventional care	C. Developing supportive mechanisms for better care
Sub-themes	A1. Experiencing the communication gap; A2. Longing for communication; A3. Communicating equals healing	B1. The nurse liaison; B2. Care beyond limits	C1. Supporting care through technology; C2. Transforming organization and care

Theme A: caring for the patient and the family

In the first main theme, aspects of patient care and family support were revealed by the nurses who participated in the study. Concepts of "burden," "desire," and "empowerment" were highlighted throughout the interviews. As such, three sub-themes were formed, namely: A1) experiencing the communication gap; A2) longing for communication; and A3) communication equals healing.

A1. Experiencing the Communication Gap

Caring for COVID-19 patients amid the restrictions on family visitation appeared to be a particularly challenging task for nurses. The lack of communication between the patient and the family caused feelings of physical and mental exhaustion. The absence of the family, the patients’ isolation, the effort to successfully meet the patient's physical needs, and the increased professional responsibilities elevated the nurses’ physical and emotional anxiety.

One of the participants, P6, said, “We are responsible for our patients... I gave too much of my personal time, as the working hours often exceeded the normal shift. I gave too much of my love and emotion to the patient who was alone. I left my personal life behind... I gave a hand... I was paralyzed suddenly after a few shifts. I woke up paralyzed, and I was out of work for three months.” 

Another participant, P2 said, “There was no one else next to them (the patients)... neither to feed them nor to help them... everything was in our hands." 

A2. Longing for Communication

Restrictive visitation policies seemed to create an increased eagerness to communicate for both the patient and the family. According to the participants’ views, the need for communication between the patient and the family during the COVID-19 era became more powerful than ever. That was because of the visitation restrictions, the uncertainty that the pandemic triggered, and the severity of the disease that the virus caused. The eagerness to communicate was vividly depicted, particularly for the older patients and for those who were in critical condition.

One participant, P8, said, “In cases of elderly patients, we were called morning, noon, and evening, and they (the family) persistently asked us about the patient... again and again if he has eaten... if we were sure that he took his medicines... We also saw the patients who transferred from the ICU to us (the ward); they needed so desperately to see their loved ones; they had missed them… so much!”

“We were dealing with parents who wanted to see their child, people who had been intubated, who had such a difficult time in the ICU and had not seen their loved ones for a long time. The first thing they were asking for was to see their family,” said P5, another participant.

A3. Communication Equals Healing

The importance of communication between the family and the patient and the family’s involvement in care was repeatedly portrayed through the nurses’ experience caring for the lonely patient. 

“... In whatever condition he (the patient) is in, the contribution of the family is enormous... Not just the family, but any person who really cares about that person... Loneliness is a disease on its own.", said P3.

Communication with the family seemed to comfort and empower the patients, the nurses, and the family members. As the patients were emotionally "healing" through meeting their relatives and gaining strength to continue, relatives and nurses strengthened their efforts to care for and support the patient.

One participant, P5, said, “The exceptions (of visitation) were very few and for specific reasons… and in these cases, I noticed that their (the patients) appearance, their face, and their mood changed completely, and they got strength and courage to get well and to try harder as they had their loved ones were next to them. This was very important for the patients who transferred from the ICU; it was proof that I am here and alive! ... It was a healing process for the patient and for the family too. And when they (the family) were seeing the patient, we earned their trust; that was rewarding.” 

Theme B: extending conventional care

The nurses’ role in supporting the COVID-19 patient, who was isolated without being able to see his/her family, was thoroughly discussed by our study participants. Interview data revealed concepts of “connecting” and "exceeding." The analysis of the data led to the formulation of two sub-themes: B1) nurse liaison, and B2) care beyond limits.

B1. The Nurse Liaison

As the restriction of visitation increased the patients’ loneliness and the need for communication, nurses found themselves undertaking new roles. In many cases, nurses relayed the patient's messages to the family and vice versa. Bonding relationships developed between nurses and patients, and at the same time, the nurses became the patients’ liaison with the outside world.

“We were trying to bring them into contact with the outside world... families brought us things to give them: clothes, water, food... We had treated mother and daughter in different wards, and they were asking us, "Is my daughter next door okay?" Although they were talking over the phone, they wanted us to tell them too”, said P1.

Nurses were next to the patient, empathizing with them and supporting them in difficult moments. Informing and helping them to communicate with the outside world, breaking bad news, and conveying the patients’ last wishes to the family were some of the tasks that nurses had to undertake. 

“I remember a dying patient. She asked me to give a message to her niece, her only relative, so I did. When her niece came later on to collect her personal belongings, I told her what her aunt told me. It was a very emotional moment. You convey a message from a person who has passed away, and you know that this is the last thing she wants to say to her only relative… an unpleasant task... what she said to me… I don't know if she would say it to anyone else," said P5.

B2. Care Beyond Limits

According to the nurses' descriptions, the changes that occurred due to the absence of the family from the patients’ daily care strengthened the nurse-patient relationship. The patients developed trusting relationships with the nurses, and the nurses supported lonely patients by providing care beyond their limits. They became the patients’ family members, their closest relative, and their closest friends.

“Many times, it happened to stand next to the patient, not as a nurse but as a daughter, as a friend... Not to provide care, nor to give medication, but to be next to him to sit there and talk and support him," said P5.

One of the participants, P2, said, “It was a case, a man who was going to die… I was trying to... to be there for him as if I were his daughter (moved)... to be close to him, as if he were my father... to caress him, to say words... to encourage him... because we were there as if we were... their only world”.

A major issue that nurses faced was the patient, who was dying alone. Feelings of distress and agony were reported by the nurses who cared for the patients who died without being able to say goodbye to their loved ones. Similarly, in certain cases, the patients experienced an inability to attend the funerals of their relatives. The nurses supported not only the lonely patient but also the family members who had to deal with feelings of loss and bereavement as they were unable to see their loved ones even after their deaths.

“I've seen them (the patients) crying, struggling a lot... They could not have the emotional support of their relatives. This emptiness, this loneliness, was communicated to us... “I am here alone... I will die alone." We lived in the constant agony of death... So, we had to comfort them, talk to them, explain to them... I was trying to give them courage. and this distressed me so much...” said P9.

“I remember an incident... A woman... She was in our ward, but she was fine... Her mother died from COVID, and she was crying and screaming because she couldn't come out and bury her. She couldn’t go to her mother’s funeral," said P3.

One participant, P11, said, “We informed the family that the patient has died and they had not seen him at all... And for the family, this was a difficult situation. They could not even see him after his death. The patients who were dying alone were a big social problem... and those who were left behind were full of guilt that they failed to stand by their people in those moments."

The participants suggested that none of the patients at their end of life should be alone and that family members should retain their right to say goodbye to their loved ones.

“When a patient is dying, the presence of the family is important for the patient... It’s very sad to die alone. And for the family… how can they manage the loss when they are absent?” said P10.

Theme C: developing supportive mechanisms for better care

Experiencing the phenomenon of visitation restriction and caring for lonely patients led nurses not only to develop new practices but also to adopt new supportive mechanisms for improving nursing practice and for involving the family in the patients’ care. Data analysis revealed concepts of "technology" and "management," and two sub-themes emerged: C1) supporting care through technology, and C2) transforming organization and care.

C1. Supporting Care Through Technology

According to the nurses’ experience, care was sustained and improved through the use of technology. Mobile phones and tablets were part of daily routine care, as these were the means of communication between patients and their families. Further to this, the quality of nursing care was sustained and enhanced through the extended use of the intranet and intercom systems that operated in certain areas. The use of cameras, remote monitoring, and communication systems within the hospital has facilitated the nursing work and supported the effective monitoring of patients. The integration of technology into nursing care was deemed not only necessary but extremely beneficial for the effectiveness of nursing care.

“We had cameras and intercom facilities... so we were able to communicate with those patients who were in critical condition. With older patients, we could monitor and control the situation; we could give safe and good care to our patients," said P4.

“The young patients used their phones or Skype to speak with their relatives; most of them had their personal computers (PCs). The difficult thing was the isolation in the room... Thank goodness the technology helped us deal with it," said P8.

The use of technology was not without limitations. Many challenges were faced, mainly by the older patients. The nurses’ support in solving problems appeared to be important for patients’ satisfaction with the care received.

One participant, P4, said, “We had a gentleman who couldn't manage his mobile phone, and whenever we entered the room, he was asking for our help to call his wife... I did it every time I walked in... and I wanted to do it... So the patient was happy, enjoying a chat with his wife even over the phone... Communication and care were efficient."

C2. Transforming Organization and Care

Managing COVID-19 patients has been a challenge for the nursing staff, as the rapid organizational changes and the implementation of novel protocols revealed the necessity to resolve a variety of organizational problems and deficiencies. Nurse participants stressed that the provision of quality nursing care in times of healthcare crises requires adequate and appropriate staffing, clarity and suitability in task allocation, and a competent interprofessional team.

One of the participants, P3, said, “Yes, we could work better if we had more staff if the nurse-patient ratio was better... If our professional responsibilities and tasks were focused on caring for the patients... It is not possible for nurses to have other duties... to be an electrician, a ward assistant, or a psychologist.”

“For difficult cases, for the patients who cannot meet their relatives, I think that there should be an interprofessional team that can handle the patient’s needs. Social workers, psychologists, and nurses should work together to resolve such issues... in a way that we cannot do it alone," said P10.

Staff development in terms of gaining expertise and training on psychological and social support mechanisms was regarded by the participants as a significant action that should be preserved by the hospital’s top management.

“We have not learned to do it (psychosocial support), and we have not been trained in it, so it makes it difficult for us to manage all these... and the hospital, managers, should support us and give us the opportunity to get some knowledge and some training about it... to ensure competent services so that patients and their families receive good and specialized care," said P7.

Finally, nurses recommended a more flexible visitation policy, as this was regarded as beneficial to the patient and the family.

One participant, P1, said, “Now that we're all a bit more familiar with the virus, more knowledgeable, and that we've learned how to protect ourselves, I think they could implement it in clinics (a flexible visitation policy). This would help them both (the patients and the family)."

## Discussion

The purpose of this study was to explore nurses' views on caring for lonely patients during the COVID-19 pandemic. Our findings highlighted the necessity of adopting a flexible family visitation policy, which will enable family members, nurses, and patients to work together toward a good therapeutic outcome. The communication gap due to family visitation restrictions, the demanding responsibilities, and the new roles that nurses had to adopt in caring for lonely patients were underscored. Moreover, the changes in care resulting from the rapid transformation of health systems imposed by the pandemic were highlighted.

Caring for the patient and the family

Caring for the patients and their families during the pandemic challenged clinical practice in various ways. Lack of communication between the patients and their loved ones caused physical and mental exhaustion for the nursing staff and increased the responsibilities of caring for lonely patients. This evidence is supported by relevant research that underscores the physical and emotional burden experienced by nurses due to the nature of the disease, the restrictive measures imposed, the highly demanding care, and the difficulties in meeting the patient's needs [6,23]. In a similar study, nurses reported symptoms of post-traumatic stress disorder after caring for COVID-19 patients [24]. In addition, isolation and separation from the family caused negative emotions in patients, such as sadness, anxiety, despair, and fear of dying alone [5]. These appeared to have an adverse impact on nurses’ work and patients’ well-being, while the involvement of the family in care and decision-making was considered beneficial for the patient’s outcomes [25]. Communication and family involvement in care are essential for supporting the patients’ self-management and self-care. The need for family-centered care is highlighted as a considerable factor for the patients and the family’s well-being [26]. Diaz-Agea et al. [27] pointed out the negative impact that the family visitation restrictions had on COVID-19 patients and their families. They further state that the family’s contribution to patient care is important, while patient-family communication supports the caring process [27].

Extending conventional care

Our findings highlighted the new roles that nurses had to undertake while caring for lonely patients. Nurses became the patients’ liaison with the outside world. They provided care beyond limits, becoming, in some cases, the closest relative of the patient, supporting the patients who were dying alone and the families who were not able to say goodbye to their loved ones. Similar findings are reported in the relevant literature, as the absence of the family created new patterns in nurse-patient communication and developed strong bonding relationships between caregivers and patients [28-30]. Jia et al. demonstrated that family visitation restrictions directed nurses towards offering a wide range of support to their isolated patients and adopting a mediating role between the patients and their families [31]. This included communicating routine information from the patient to the family or even conveying the patient's last wishes to their loved ones.

Deep and intense feelings of sadness and distress were experienced by the nurses as they were confronted with the patient, who was dying alone. This issue was discussed in the relevant literature by underscoring the need for the family to be next to the dying patient and the need for the dying patient to be surrounded by his/her relatives [30-33]. In consistency with the findings of the present study, Feder et al.'s study highlights the importance of sharing feelings of loss and mourning in cases where relatives were unable to be next to their loved ones even after death [34]. Despite the physical and emotional burden that this issue involved, nurses demonstrated high levels of empathy, professional responsibility, and dedication by standing next to the patients and their families in difficult moments [12, 35, 36]. To a further extent, the patients’ families appeared to have a substantial role in the nurses' well-being and satisfaction, especially when caring for terminally ill patients. By identifying ways of involving family in patients’s care, nurses’ fulfillment and sense of worth can be enhanced [37].

Developing supportive mechanisms for better care

According to our study’s findings, technology and administrative interventions were regarded as supportive mechanisms for better care. According to the relevant literature, the use of technology as a means of communication between COVID-19 patients and their families is important, as it assists the effective monitoring of patients and facilitates nursing practice [38]. Relevant research highlighted the necessity of technology as a means of effective communication between patients and families during the pandemic, despite the difficulties of its utilization, mainly by older patients [7, 39, 40]. The use of technology provides a significant advantage in managing the effects of patient isolation, as it has been shown to improve the level of communication between healthcare professionals and patients, provide entertainment and information, and facilitate the communication of patients with their loved ones [41]. In addition, educating and properly informing nursing staff on the utilization of new technologies for monitoring patients' conditions is considered an important administrative priority [42].

Managing COVID-19 patients has been a challenge for the nursing staff, as the implementation of strict guidelines and protocols revealed organizational problems and shortcomings, such as staff shortage, inappropriate task allocation, and the lack of competent and well-organized interprofessional teams. In a similar study, nurses who cared for patients with COVID-19 exhibited stress and burnout due to the insufficient number of nurses working in the treatment center [43]. Relevant research evidence indicates that the empowerment and autonomy of the nursing staff are related to appropriate task allocation, which is essential for the provision of quality nursing care, especially in times of health crises [44].

The care of COVID-19 patients and their families by competent healthcare professionals was considered essential for the quality of the services rendered. Nurses referred to professional development and opportunities for expanding their knowledge to provide integrated care to their patients. Similar research evidence demonstrates the advantages of educating health professionals about supporting and providing empathetic care to critically ill patients in ICUs. These relate to encouraging the family to participate in the patient's care plan, the reduction of patients’ negative emotions caused by isolation, as well as better prognosis and satisfaction from caring for the patients and their relatives [45]. Further to this, a flexible visitation policy was regarded as essential to alleviate stress and empower the patients, their families, and the nursing staff [30].

Study limitations

This study was conducted at a single COVID-19 reference hospital in the urban region of Attica, Greece. Time restrictions, nurses’ heavy workload, and organizational transformation in the healthcare system due to the pandemic did not allow us to collect data from other sites. In this respect, the applicability of our findings should be viewed under these limitations. Further research involving multiple healthcare organizations in different geographical areas might provide more in-depth knowledge about the topic under investigation. Specifically, issues regarding family involvement in care and decision-making may differ from urban to rural areas due to cultural diversities. Similarly, the nurses’ experiences in rural, non-referral hospitals regarding the death and bereavement of the isolated COVID-19 patient and the family may differ significantly, generating new research evidence in this field of inquiry.

## Conclusions

The COVID-19 pandemic created new working conditions and different patterns of care in healthcare organizations. The care of the isolated patient, the restrictions on family visits, and the use of new technology imposed new roles and responsibilities on nurses. Maintaining a communication continuum and supporting the family’s involvement in patient care are considered to be equal to healing for lonely patients. Supporting care through technology and providing opportunities for staff development and empowerment appeared to be a necessity for providing quality care to COVID-19 patients and their families. Further research on exploring families’ and patients’ views of caring during the pandemic is recommended. These sort of data may provide a deeper understanding of the caring patterns and effective management of lonely patients in high-demand healthcare contexts.
